# Multiplex qPCR Assay for Simultaneous Detection of Three PST-Producing Dinoflagellates in the East China Sea off Southern Korea

**DOI:** 10.3390/biology15030219

**Published:** 2026-01-24

**Authors:** Jung Soo Heo, Biet Thanh Tran, Keun-Yong Kim, Sunju Kim, Seok Hyun Youn, Tae Gyu Park

**Affiliations:** 1 Bioinformatics Team, AquaGenTech Co., Ltd., Busan 48228, Republic of Korea; tranthanhbiet2502@gmail.com (B.T.T.); koby0323@hanmail.net (K.-Y.K.); 2 Major of Oceanography, Division of Earth Environmental System Science, Pukyong National University, Busan 48513, Republic of Korea; sunkim@pknu.ac.kr; 3 National Institute of Fisheries Science, Busan 46083, Republic of Korea; younsh@korea.kr (S.H.Y.); taegyupark@korea.kr (T.G.P.)

**Keywords:** *Alexandrium catenella*, *Alexandrium pacificum*, environmental DNA, *Gymnodinium catenatum*, harmful algal blooms, multiplex qPCR assay, paralytic shellfish toxin

## Abstract

Paralytic shellfish toxins (PSTs) are produced by several toxic species of the dinoflagellate genera *Alexandrium* and *Gymnodinium*, and they pose significant threats to marine ecosystems and public health. Rapid and accurate detection of harmful algal blooms (HABs) is essential for effective management. In this study, we developed a multiplex quantitative real-time PCR (qPCR) assay targeting the 28S ribosomal DNA region to simultaneously detect three PST-producing dinoflagellates, *Alexandrium catenella*, *A. pacificum*, and *Gymnodinium catenatum*, in the East China Sea off southern Korea. Species-specific primers and hydrolysis probes labeled with distinct fluorophores were validated for simultaneous detection. The standard curves showed strong linearity (*R*^2^ > 0.99) and high amplification efficiencies (95.268–99.325%). No cross-reactivity was observed among the 20 non-target microalgal species. Field application of the assay using environmental DNA (eDNA) samples collected during spring successfully detected *A. catenella* and *A. pacificum*, whereas *G. catenatum* was not detected during the survey period. This multiplex qPCR assay provides a rapid and reliable molecular tool for early detection and spatial monitoring of potentially PST-producing dinoflagellates, supporting sustainable HAB management in East Asian coastal ecosystems.

## Simple Summary

Some microscopic algae in the ocean produce strong toxins that can accumulate in shellfish and cause serious food poisoning in humans. Detecting these harmful algae early is important to protect public health and marine ecosystems, but traditional identification methods are slow and often unreliable. In this study, we developed a DNA-based test that can quickly and accurately detect three toxin-producing algae species at the same time using a single analysis. This method was tested with seawater samples collected near southern Korea and successfully detected two harmful species that are commonly found in the region. The new test is fast, sensitive, and reliable, making it useful for routine monitoring of coastal waters. By improving early detection of toxic algae, this approach can help prevent shellfish poisoning events and support safer seafood production and marine management.

## 1. Introduction

Harmful algal blooms (HABs) caused by paralytic shellfish toxin (PST)-producing dinoflagellates pose a significant and increasing threat to marine ecosystems, aquaculture, and public health worldwide [1,2]. Blooms of the toxic dinoflagellates *Alexandrium catenella*, *Alexandrium pacificum*, and *Gymnodinium catenatum* are among the most problematic harmful algal events globally because these species produce potent neurotoxins such as saxitoxins, which bioaccumulate in shellfish and cause paralytic shellfish poisoning (PSP) in humans [3,4,5]. Consequently, monitoring the occurrence and distribution of these PST-producing dinoflagellates is essential to mitigate their ecological, economic, and public health impacts.

Recently, HABs caused by PST-producing dinoflagellates have been increasingly reported in East Asian coastal waters, particularly in the East China Sea and along the Korean coast. In Korean waters, *A. catenella* and *A. pacificum* are among the dominant species, frequently detected in Jinhae–Masan Bay, Tongyeong, and other southern coastal regions [6,7,8]. Several PSP outbreaks have been documented since the early 2000s, leading to the temporary closure of shellfish harvesting areas as a precautionary public health measure [6,9]. Similarly, in Chinese coastal waters, blooms of *Alexandrium* species and *G. catenatum* have been reported in the Bohai Sea, Yellow Sea, and East China Sea, often coinciding with elevated levels of PSTs in mussels and scallops [10,11,12]. These observations highlight the increasing frequency and ecological impact of toxic dinoflagellate blooms in these areas [13,14]. Rapid and accurate identification of these toxic species is essential for effective monitoring, early warning, and timely mitigation of HABs.

However, traditional identification of dinoflagellates based on morphological characteristics observed under light microscopy is often challenging and unreliable, particularly for morphologically similar or cryptic species [15,16]. Many harmful *Alexandrium* and other dinoflagellate species exhibit considerable morphological plasticity, with diagnostic traits such as cell size, shape, and thecal plate patterns that vary according to environmental conditions, life cycle stages, and physiological states. Consequently, closely related species are frequently indistinguishable based on morphology alone. A notable example is the *A. tamarense* species complex, historically regarded as a single globally distributed species; however, molecular phylogenetic analyses have revealed that it comprises several genetically distinct yet morphologically overlapping lineages, including *A. pacificum*, *A. tamarense*, *A. fundyense*, *A. australiense*, and *A. mediterraneum* [5,17,18]. The recognition of these as distinct genetic lineages within the complex underscores the limitations of microscopy-based identification and highlights the need for alternative molecular methods capable of providing rapid, specific, and sensitive detection of harmful dinoflagellate species. In parallel, chemical analytical techniques such as liquid chromatography–tandem mass spectrometry (LC–MS/MS) are widely used to quantify PSTs directly in water and shellfish tissues, providing definitive confirmation of toxin presence, but without species-level resolution of the causative organisms [19].

To overcome the limitations of morphology-based identification and toxin-only measurements, molecular techniques have been widely adopted for the detection and quantification of harmful dinoflagellates [1,20]. Conventional polymerase chain reaction (PCR) enables species-specific amplification of diagnostic DNA fragments, whereas quantitative real-time PCR (qPCR) offers enhanced sensitivity, specificity, and the ability to quantify target species in mixed environmental samples. Other approaches, such as digital PCR (dPCR) and metabarcoding using next-generation sequencing (NGS), have also been applied for HAB monitoring. dPCR allows the absolute quantification of target genes without the need for standard curves and provides superior tolerance to PCR inhibitors, making it suitable for detecting low-abundance or degraded environmental DNA (eDNA) [21]. Metabarcoding, on the other hand, enables comprehensive community profiling and simultaneous detection of multiple algal taxa, facilitating the discovery of novel or co-occurring species in bloom samples [22]. Nevertheless, qPCR remains the standard and most commonly used molecular method for routine HAB monitoring owing to its high sensitivity, quantitative capability, short assay time, and cost-effectiveness [23,24].

Recent advances in multiplex qPCR have greatly enhanced the molecular detection efficiency by enabling simultaneous amplification and detection of multiple target species within a single reaction [24,25]. This approach utilizes several species-specific primer and hydrolysis probe sets, each labeled with a distinct fluorescent dye, allowing multiple harmful algal species to be detected in the same sample in one assay [25]. Compared with microscopy-based identification, which is labor-intensive, time-consuming, and highly dependent on taxonomic expertise, multiplex qPCR enables rapid, objective, and species-specific detection with substantially greater sensitivity, particularly for low-abundance or cryptic taxa in environmental samples [23,24]. DNA metabarcoding, while providing comprehensive community-level information, typically requires longer processing times, entails higher sequencing and bioinformatics costs, and often yields only semi-quantitative data [22]. In contrast, multiplex qPCR allows rapid and cost-effective quantification of predefined target species with high analytical sensitivity [25,26]. Accordingly, for monitoring programs focused on the early detection and routine surveillance of known harmful dinoflagellate species, multiplex qPCR represents a practical and efficient complementary approach to both microscopy and metabarcoding. Eckford-Soper and Daugbjerg [20] demonstrated the potential of this approach by developing a high-throughput multiplex qPCR assay based on hydrolysis probe technology for the simultaneous detection of four globally distributed HAB-forming species: *A. tamarense*, *Karenia mikimotoi*, *Karlodinium veneficum*, and *Prymnesium parvum*.

In this study, we developed and validated a multiplex qPCR assay targeting the 28S ribosomal RNA gene (rDNA) region for the simultaneous detection of three PST-producing dinoflagellates: *A. catenella*, *A. pacificum*, and *G. catenatum*. Species-specific primers and hydrolysis probes labeled with distinct fluorophores were designed to ensure sensitivity and specificity. The assay was performed using laboratory-cultured strains and eDNA samples collected from the East China Sea in southern Korea. This multiplex qPCR assay provides a rapid, reliable, and medium-throughput molecular tool for the early detection and monitoring of PST-producing dinoflagellates, contributing to improved HAB surveillance and seafood safety in East Asian coastal ecosystems.

## 2. Materials and Methods

### 2.1. Seawater Sample Collection and eDNA Extraction

**Figure 1:**
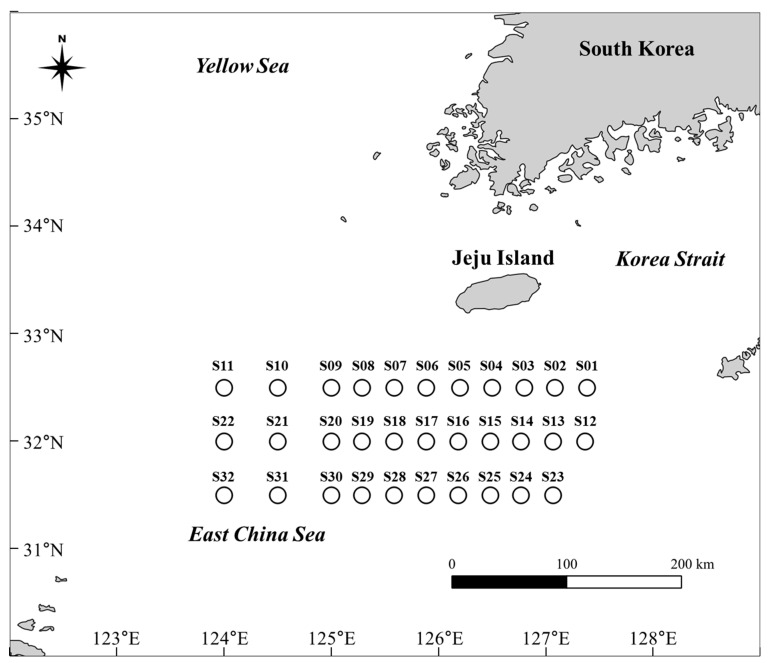
Seawater sample collection sites at 32 stations (S01–S32) in the East China Sea off southern Korea. Samples were used for environmental DNA monitoring of *Alexandrium catenella*, *Alexandrium pacificum*, and *Gymnodinium catenatum*.

The study area was the East China Sea, which is a transitional zone between the Korea Strait and the Yellow Sea, and is an area associated with recurrent toxic algal blooms [5,13]. During the sampling period in late April, hydrographic data collected by the National Institute of Fisheries Science (NIFS; https://www.nifs.go.kr, accessed on 25 October 2025) aboard the Tamgu 3 research cruise indicated typical springtime conditions in the East China Sea off southern Korea. Water temperatures ranged from approximately 12.4 to 20.7 °C, salinity values ranged from 32.1 to 34.6 psu, and dissolved oxygen concentrations varied between 4.25 and 8.10 mL L^−1^ across sampled sites. A total of 32 seawater samples (2 L) were collected in sterile disposable plastic bottles by the NIFS of South Korea as part of a fixed research cruise schedule of Tamgu 3 from April 26 to May 3, 2021 (Figure 1, Supplementary Table S1). The samples were vacuum-filtered through glass microfiber filters (Grade GF/C, 47 mm; Whatman, Marlborough, MA, USA). Each filter was carefully folded using sterile forceps, placed in a 2.0 mL microtube, and stored in a cooled icebox with polyethylene bags containing ice shielded from light exposure. The filters were then directly transported to the laboratory and stored at −70 °C until further use.

For eDNA extraction, each filter was mechanically disrupted using an Omni Bead Ruptor 12 Bead Mill Homogenizer (OMNI International, Kennesaw, GA, USA) and extracted using the DNeasy Blood & Tissue Kit (Qiagen, Hilden, Germany) following the manufacturer’s protocol, and then eluted in 50 μL of sterile distilled water. The extracted eDNA was immediately stored at −20 °C for further analysis.

### 2.2. Design of Primers and Hydrolysis Probes

All available 28S rDNA sequences of *A. catenella*, *A. pacificum*, *G. catenatum* and their closely related species were retrieved from GenBank (https://www.ncbi.nlm.nih.gov/, accessed on 17 October 2025) and aligned using ClustalW in BioEdit 7.2 [27]. The sequence matrix was manually corrected by visual inspection to ensure alignment accuracy (Supplementary Figure S1). Primers and hydrolysis probes were regarded as being specific for the three PST-producing dinoflagellates if they contained at least two total mismatches to non-target species, including at least two mismatches within the last five base pairs of the 3′ end for both forward and reverse primers. Candidate primers and hydrolysis probes were evaluated for specificity, GC content, and melting temperature (*T*_m_), and screened for potential secondary structures using Sequence Manipulation Suite ver. 2 (https://www.bioinformatics.org/sms2/, accessed on 25 October 2025) [28]. To ensure target specificity while minimizing cross-reactivity, the final multiplex qPCR primer-probe set was designed based on species-specific variable regions. Each hydrolysis probe was labeled with a distinct fluorophore-quencher pair to enable simultaneous detection in a single reaction. The primer concentrations and thermocycling conditions were adjusted to ensure efficient co-amplification of the three target species.

### 2.3. Multiplex qPCR Parameters

Multiplex qPCR optimization was performed by testing different reagent, primer, and hydrolysis probe concentrations as well as varying annealing temperatures. All qPCR reactions were conducted in 96-well plates using a QuantStudio 5 Real-Time PCR System (Thermo Fisher Scientific Inc., Waltham, MA, USA) under the following conditions: an initial activation step at 95 °C for 2 min, followed by 40 amplification cycles consisting of denaturation at 95 °C for 15 s and annealing/extension at 60 °C for 45 s. The optimized multiplex qPCR reaction was prepared in a 20 μL total volume, consisting of 10 μL GoTaq Probe qPCR Master Mix (Promega, Madison, WI, USA), 0.25 μM of each primer, and 0.125 μM of each hydrolysis probe, synthesized by Macrogen Inc. (Seoul, Republic of Korea). All qPCR assays were manually set up in triplicate and included no-template reactions as negative controls. Data were analyzed using QuantStudio Design & Analysis Software 1.5.2 (Thermo Fisher Scientific Inc.). Each standard curve was obtained from a single experimental run and each dilution was analyzed in triplicate. The mean ± standard deviation (SD) was calculated from three technical replicates.

### 2.4. Standard Curve Generation and Efficiency Assessment

qPCR assay efficiencies were determined using standard curves generated from 10-fold serial dilutions of three plasmid DNAs synthesized and cloned by Bioneer Inc. (Daejeon, Republic of Korea). Partial 28S rDNA fragments containing all complementary binding sites of primers and hydrolysis probes for each target species were inserted, spanning 10^7^–10^1^ copies per reaction (rxn^−1^). Each dilution was tested in technical triplicate to ensure accuracy, and a no-template negative control using nuclease-free water was included. The plasmid DNAs were quantified using a Qubit 4.0 fluorometer (Thermo Fisher Scientific Inc.), and their integrity was verified by agarose gel electrophoresis. Standard curves were generated by plotting the cycle of quantification (C_q_) values against the log-transformed plasmid DNA copy number. The plasmid-equivalent copy numbers were calculated based on the standard curve using the equation C_q_ = m × log_10_(copies) + b, where C_q_ is the quantification cycle value, m is the slope of the standard curve, b is the y-intercept, and the copies represent the DNA copy number rxn^−1^. The amplification efficiencies, calculated from the slope of the standard curve (E = (10^(−1/m)^ − 1) × 100), ranged between 90 and 110%, with a correlation coefficient (*r*^2^) value exceeding 0.99, confirming high assay reliability. The abundances of *A. catenella*, *A. pacificum*, and *G. catenatum* in 2 L environmental seawater samples were estimated based on plasmid-equivalent copy number estimates from triplicate qPCR reactions and converted to copies L^−1^ by scaling to the total DNA elution volume (50 µL), correcting for the template volume per reaction (1 µL), and normalizing to the filtered seawater volume (2 L) using the following equation: copies L^−1^ = (copies rxn^−1^ × elution volume [50 µL]/template volume [1 µL])/filtered seawater volume [2 L].

### 2.5. Specificity Test for Multiplex qPCR Assay

The specificities of the newly designed primers and hydrolysis probes were evaluated against three target species and 20 non-target phytoplankton species (Supplementary Table S2). They were harvested during the exponential growth phase, centrifuged at 4000× *g* for 15 min, and the resulting pellets were used for genomic DNA (gDNA) extraction using an APrep Total DNA kit (AP Bio Co., Ltd., Namyangju, South Korea) following the manufacturer’s protocol. The concentration and purity of the extracted gDNA were assessed using a NanoDrop One spectrophotometer (Thermo Fisher Scientific, Inc.). The extracted gDNAs were diluted to 20 ng µL^−1^ in the TE buffer and stored at −20 °C until use.

Specificity testing was performed using 20 ng of gDNA from 20 non-target species and the three target species as templates in independent multiplex qPCR assays. Each template DNA sample was tested in a single reaction using the optimized multiplex qPCR conditions, and all reactions were conducted within a single qPCR run. Fluorescence signals were recorded for each reaction, and amplification was observed only for the intended targets, with no amplification detected for any non-target species or negative controls (Cq > 40). 

### 2.6. Multiplex qPCR Assay on Environmental Seawater Samples

The eDNA extracted from 32 seawater samples collected in the East China Sea off southern Korea was used as a template (1 μL) for each qPCR assay under optimized multiplex qPCR conditions to detect and quantify the three target species. The amplification efficiency (E), specificity, and sensitivity of the multiplex qPCR assay were evaluated based on the C_q_ values, with detection considered positive when the C_q_ values crossed the automatically determined threshold set by the QuantStudio 5 system and remained within the linear range of the standard curve. The copy number of rxn^−1^ was determined using a standard curve generated from 10-fold serial dilutions of plasmid DNA from each target species.

## 3. Results

### 3.1. Validation of Primers and Hydrolysis Probes

Species-specific qPCR primers and hydrolysis probes complementary to the 28S rDNA region were designed for three target species: *A. catenella*, *A. pacificum*, and *G. catenatum* (Table 1), with expected amplicon sizes of 215, 245, and 233 bp, respectively. The hydrolysis probes were labeled with FAM, HEX, and CY5 and with BHQ1, MGB Eclipse, and BHQ3 quenchers to ensure efficient fluorescence detection. The nearest-neighbor *T*_m_ ranged from 55.5 °C to 63.5 °C.

### 3.2. Standard Curve and Efficiency Validation

**Figure 2:**
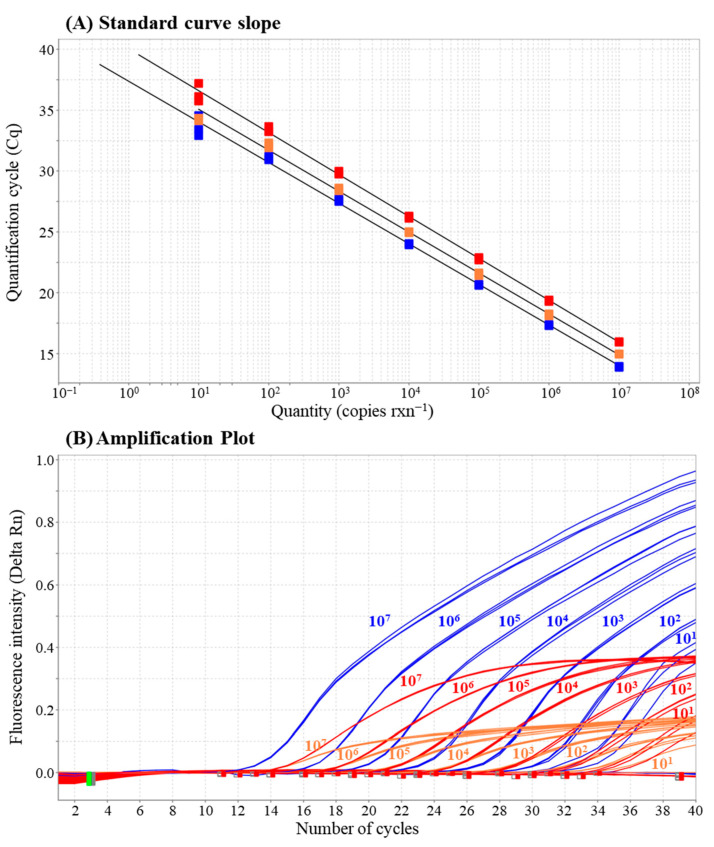
(**A**) Standard curves and amplification plots for the multiplex quantitative real-time PCR assay targeting *A. catenella* (blue), *A. pacificum* (red), and *G. catenatum* (orange). (**B**) Standard curves were generated using 10-fold serial dilutions of plasmid DNAs, showing strong linearity (R^2^ > 0.99) and amplification efficiencies between 95% and 99%.

The qPCR standard curves for *A. catenella*, *A. pacificum*, and *G. catenatum* were generated using 10-fold serial dilutions of plasmid DNAs spanning 10^7^–10^0^ copies of rxn^−1^, with each dilution tested in triplicate (Figure 2A). Linear regression equations yielded the equations y = −3.338x + 37.378 for *A. catenella*, y = −3.441x + 40.033 for *A. pacificum*, and y = −3.364x + 38.448 for *G. catenatum* (Figure 2B). The slopes (−3.338, −3.441, and −3.364) yielded R^2^ values of 0.997, 0.998, and 0.997, respectively, confirming a strong linear relationship between Cq values and log-transformed DNA copy numbers for all three target species. The amplification efficiencies calculated from the slopes were 99.325% for *A. catenella*, 95.268% for *A. pacificum*, and 98.278% for *G. catenatum*, falling within the optimal range (90–110%) and indicating reliable and efficient amplification. The limit of detection (LOD) for each assay was 10 copies rxn^−1^.

### 3.3. Specificity of Multiplex qPCR Assay

**Figure 3:**
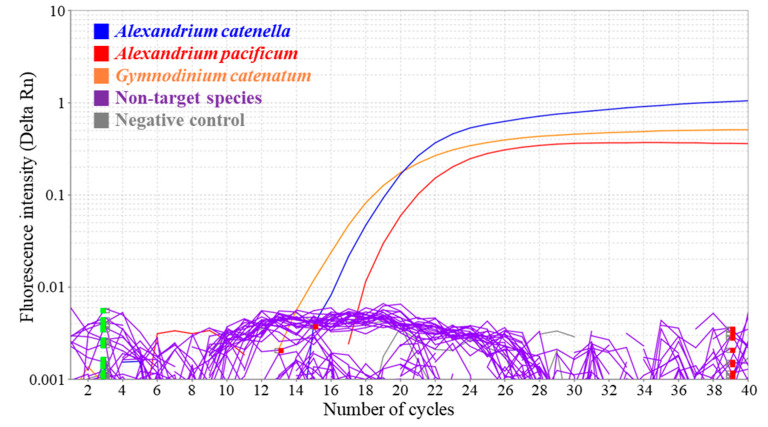
Detection specificity of the multiplex quantitative real-time PCR (qPCR) assay for *A. catenella*, *A. pacificum*, and *Gymnodinium catenatum*. Specificity was evaluated using genomic DNA (20 ng µL^−1^) from the three target species and 20 non-target microalgal species (see Supplementary Table S2). No amplification was observed in any non-target species or in the negative control, confirming the high specificity of the multiplex qPCR assay. Green and red boxes indicate software-defined baseline regions for different fluorescence channels.

The specificity of the multiplex qPCR assay was evaluated for three target species, *A. catenella*, *A. pacificum*, and *G. catenatum*, along with 20 non-target species (Supplementary Figure S2, Table S2) and one negative control. The amplification curves exhibited clear sigmoidal patterns with mean C_q_ values of 18.340, 19.359, and 16.711, respectively (Figure 3), indicating successful and specific amplification. In contrast, no amplification was observed for the non-target species or the negative control without C_q_ values exceeding 40 in all cases, confirming the absence of non-specific amplification or contamination. These results validated the high specificity of the multiplex qPCR assay using the newly designed primers and hydrolysis probes for detecting *A. catenella*, *A. pacificum*, and *G. catenatum*.

### 3.4. Multiplex qPCR Assay of eDNA Samples

Table 2 shows the C_q_ values and DNA copy numbers obtained from multiplex qPCR assays of the 32 eDNA samples for the detection and quantification of *A. catenella*, *A. pacificum*, and *G. catenatum*. Among the 32 sampling stations, *A. catenella* was detected at 15 stations (S05, S06, S07, S08, S09, S17, S18, S19, S20, S21, S22, S24, S26, S28, and S29), whereas *A. pacificum* was detected at 19 stations (S04, S05, S06, S07, S08, S15, S16, S17, S18, S19, S20, S21, S22, S23, S24, S25, S26, S28, and S29). In contrast, *G. catenatum* was not detected in any of the analyzed samples, which is consistent with its reported low abundance during spring.

The highest mean plasmid-equivalent copy number of *A. catenella* was observed at station S29 (9086.7 ± 1727.2 copies L^−1^), followed by S28 (2015.6 ± 192.6 copies L^−1^) and S26 (1538.9 ± 178.6 copies L^−1^). Lower concentrations ranged from 8.8 to 993.4 copies L^−1^. Similarly, *A. pacificum* exhibited its highest abundance at S22 (10,434.0 ± 1211.1 copies L^−1^), followed by S07 (3666.1 ± 762.1 copies L^−1^) and S05 (3642.7 ± 761.0 copies L^−1^). The lowest DNA copy numbers were detected at S18 (58.9 ± 51.4 copies L^−1^), S24 (227.2 ± 44.1 copies L^−1^), and S15 (223.0 ± 115.9 copies L^−1^). Detailed results, including C_q_ values, quantification values (copies rxn^−1^), and total volume-equivalent concentrations (copies L^−1^) for each station and target species, are provided in Supplementary Table S3.

## 4. Discussion

In this study, we developed and validated a multiplex qPCR assay for the simultaneous detection and quantification of three PST-producing dinoflagellates: *A. catenella*, *A. pacificum*, and *G. catenatum*. Development of multiplex qPCR assays requires selecting an appropriate molecular marker; rRNA gene regions are widely favored for their high copy number, extensive reference databases, and sensitivity in species-specific detection [15,23]. The 18S rDNA is highly conserved for higher-level taxonomy but lacks variability for discriminating closely related *Alexandrium* species [18]. The ITS region offers better resolution for the *A. tamarense* complex but suffers from intragenomic polymorphism and length variants that hinder multiplex primer/probe design [29,30]; in contrast, the 28S rDNA balances variability and stability for reliable discrimination of paralytic shellfish toxin-producing dinoflagellates [24,31,32].

Targeting the 28S rDNA region enabled accurate discrimination among *A. catenella*, *A. pacificum*, and *G. catenatum*, while maintaining assay efficiency. Specificity was further confirmed through experimental testing against 20 non-target phytoplankton taxa, including chlorophytes, haptophytes, diatoms, cyanobacteria, and other dinoflagellates (e.g., *Effrenium voratum* and *Prymnesium parvum*), with no cross-amplification observed. The non-target specificity panel included phylogenetically diverse phytoplankton taxa; however, additional members of the *A. tamarense* species complex and closely related *Gymnodinium* species were not available for experimental testing. Specificity against these closely related taxa was ensured through careful selection of diagnostic regions within the 28S rDNA, with primers and hydrolysis probes designed to contain multiple sequence mismatches relative to non-target species. 

The eDNA analysis of 32 field samples from the East China Sea off southern Korea revealed the presence of *A. catenella* and *A. pacificum* at multiple stations, with the highest abundances at S29 and S22, respectively. The observed distribution patterns were consistent with previous reports of these species in Korean and Chinese coastal waters [5,13]. In contrast, *G. catenatum* was not detected, which is more likely attributable to ecological and temporal constraints. Although *G. catenatum* cysts are widely distributed in the coastal seas of China, including the East China Sea, cyst germination alone is insufficient to initiate blooms, which are instead constrained by environmental factors [33,34]. In addition, *G. catenatum* is an obligate coastal species whose bloom development depends on favorable hydrographic conditions and land-derived nutrient inputs [33,34]. Growth of *G. catenatum* is known to occur primarily at relatively warm temperatures (approximately 20–25 °C), with markedly reduced growth observed below 15 °C under experimental conditions, indicating that summer and early autumn conditions are most favorable for vegetative population development [33,35]. This temperature dependence is in agreement with field observations from Korea, where no PSP outbreaks attributed to *G. catenatum* have been reported and detections of vegetative cells have been seasonally restricted [36]. In the present study, springtime hydrographic conditions at offshore stations, with water temperatures ranging from approximately 12.4 to 20.7 °C, were likely suboptimal for sustained vegetative growth of this species. Accordingly, future monitoring efforts should extend sampling across multiple seasons and target narrower temperature windows to more effectively capture the occurrence of *G. catenatum* and further evaluate the field performance of the multiplex qPCR assay for this species. 

It should also be noted that detection of *A. catenella* or *A. pacificum* DNA does not necessarily indicate the presence of toxigenic strains or active PST contamination in water or shellfish, and therefore molecular detection alone cannot be used to confirm toxin risk [37,38]. Accordingly, the multiplex qPCR assay developed here is best viewed as an early-warning and screening tool for the presence of potentially PST-producing taxa rather than a direct measure of toxicity [37,38,39]. qPCR-derived copy numbers are best interpreted as indicators of presence and relative spatial distribution rather than absolute biomass or PST risk [38,39]. Within this context, the detection of *A. pacificum* at a greater number of stations than *A. catenella* may reflect broader spatial distribution during the sampling period rather than true numerical dominance. Alternatively, differences in cellular rDNA copy number, physiological condition, or DNA yield per cell may also contribute to higher detection frequency. In contrast, *A. catenella* was detected at fewer stations but occasionally at higher copy numbers, suggesting a more patchy distribution potentially associated with localized population development. For comprehensive threat evaluation, molecular monitoring should be combined with quantitative toxin analyses in water and shellfish (e.g., LC–MS/MS) and, where possible, toxicity screening of local strains. Distinguishing among these possibilities will require future studies integrating molecular data with microscopy-based cell counts and toxin measurements across seasons.

Although the present study did not include spiking of field samples with known quantities of target DNA or the use of an internal positive control to explicitly test for PCR inhibition, several results suggest that inhibition was minimal in the analyzed samples. All assays exhibited high and consistent amplification efficiencies (95–99%), strong linearity of standard curves (R^2^ > 0.99), and reliable amplification of target species across a wide range of environmental concentrations, including detections near the limit of quantification. Successful detection of multiple target species in the same environmental samples argues against the presence of strong, systematic inhibition. In addition, template DNA was diluted prior to qPCR analysis, a common approach to further mitigate residual inhibition in eDNA workflows [23,33]. Nevertheless, the absence of an explicit inhibition control represents a methodological limitation, as seawater eDNA can contain compounds (e.g., salts, organic matter, or humic substances) that may partially inhibit PCRs [20,24]. Future applications of the assay would benefit from the inclusion of internal positive controls or DNA spike-in experiments to directly assess and correct for potential PCR inhibition, thereby further strengthening the robustness of the assay for routine environmental monitoring.

Environmental DNA–based monitoring is inherently challenged by low target abundance, DNA degradation, and spatial heterogeneity, all of which can introduce variability in detection and quantification [40]. Although the multiplex qPCR assay was validated using plasmid standards and environmental seawater samples, its performance under conditions of extremely low target abundance or highly degraded eDNA—common in natural marine environments—was not explicitly tested in this study. Consistent with these challenges, some field samples exhibited relatively high variability among replicates (e.g., station S06: 142.61 ± 148.79 and 31.32 ± 27.18 copies L^−1^), which likely reflects stochastic effects associated with low DNA concentrations near the detection limit, patchy distribution of eDNA in seawater, and natural environmental heterogeneity rather than assay instability [32,34,40]. Despite this variability, overall detection patterns across stations and reproducible amplification performance support the reliability of the assay. Additional testing under environmentally challenging conditions, including low-abundance targets, degraded DNA, and increased replication across seasons, would help improve quantitative confidence and support broader application of the assay in monitoring programs. In addition, assay validation in this study was limited to seawater eDNA, and its direct applicability to other matrices relevant to PST surveillance, such as shellfish tissues or sediment cysts, remains to be evaluated.

Although the limit of detection of the multiplex qPCR assay was determined to be 10 copies per reaction, direct translation of gene copy numbers to regulatory action thresholds is not straightforward, as shellfish harvesting closures are based on measured PST concentrations rather than algal abundance alone. Nevertheless, previous molecular monitoring studies in Korean coastal waters have shown that toxic *Alexandrium* can be reliably detected at cell densities on the order of 10^2^–10^3^ cells L^−1^, levels that are below or near the practical detection limits of routine microscopy and can precede measurable toxin accumulation in shellfish [21,41]. These findings indicate that the detection limit achieved in the present study is ecologically relevant for early warning applications, enabling the identification of toxic *Alexandrium* populations at sub-bloom stages. Future work integrating molecular abundance data with concurrent shellfish toxin measurements will be necessary to establish quantitative relationships between gene copy numbers, cell densities, and PST risk thresholds used in management programs.

The multiplex qPCR assay developed in this study has several advantages over conventional PCR methods. It reduces the analysis time and use of reagents while maintaining high accuracy, making it suitable for large-scale environmental monitoring programs. Furthermore, the assay can be integrated with eDNA-based surveillance systems, enabling early detection of PST-producing dinoflagellates before bloom formation [13]. Applying this approach will enhance the early warning capabilities for HABs and mitigate the ecological and economic impacts associated with PSP outbreaks. Future studies should broaden this multiplex strategy to include other toxic dinoflagellates and combine molecular detection with toxin quantification for comprehensive HAB risk assessment.

## 5. Conclusions

This study was conducted to develop a robust and efficient multiplex qPCR assay for the simultaneous detection and quantification of three PST-producing dinoflagellate species, *A. catenella*, *A. pacificum*, and *G. catenatum*, in the East China Sea off southern Korea. The assay exhibited high sensitivity, specificity, and reliable performance in both laboratory and environmental samples, providing clear advantages for the molecular monitoring of harmful algal bloom (HAB) species. Field application revealed the presence of *A. catenella* and *A. pacificum* across multiple offshore stations, while *G. catenatum* was not detected during the spring sampling period. The developed assay provides a valuable molecular tool for the early detection and routine monitoring of PST-producing dinoflagellates, thereby supporting effective HAB management in East Asian coastal ecosystems.

## Figures and Tables

**Table 1:** List of species-specific forwards (f) and reverse (r) primers, along with hydrolysis probes (p) designed for multiplex quantitative real-time PCR assay targeting the 28S rDNA region of *Alexandrium catenella* (Aca), *Alexandrium pacificum* (Apa), and *Gymnodinium catenatum* (Gca).

Organism	Primer Name	Primer Sequence (5′ → 3′)	Fluorescent Dye	Quencher	Nearest Neighbor *T*_m_ (°C)	Amplicon Size (bp)
*Alexandrium catenella*	Aca-28S-0430f	TTGATTTGCTTGGTGGGAG	-	-	58.2	215
	Aca-28S-0627r	CCAAGCACAGGAACACAC	-	-	59.1	
	Aca-28S-0451p	CAAAGCTCTTGTCAAGCAAGTGCAA	FAM	BHQ1	61.5	
*Alexandrium pacificum*	Apa-28S-0427f	GACTTGATTTCCTCAGTGAGATTGTA	-	-	58.5	245
	Apa-28S-0652r	CATGTAAGCCCAAGGGAGAA	-	-	60.1	
	Apa-28S-0468p	AATTATTGCACTTGCAGCCAAAACCCA	HEX	MGBEclipse	62.7	
*Gymnodinium catenatum*	Gca-28S-0500f	GGCTGTGATTTGTTCGGT	-	-	57.7	233
	Gca-28S-0711r	TAGAACCATTTCGTCACCATTA	-	-	55.5	
	Gca-28S-0657p	CTTCGTGTTGCGTGCTCTGG	CY5	BHQ3	63.5	

**Table 2:** Multiplex quantitative real-time PCR quantification of environmental DNA from 32 seawater samples for detections of *A. catenella* (Aca), *A. pacificum* (Apa), and *G. catenatum* (Gca).

Station	Volume (mL)	Mean Quantification Cycle Value (C_q_ ± Standard Deviation)			Mean Plasmid-Equivalent Copy Number (Copies L^−1^ ± Standard Deviation)		
		Aca	Apa	Gca	Aca	Apa	Gca
S01	2000	ND	ND	ND	0.0	0.0	0.0
S02	2000	ND	ND	ND	0.0	0.0	0.0
S03	2000	ND	ND	ND	0.0	0.0	0.0
S04	2000	ND	36.83 ± 0.76	ND	0.0	248.21 ± 107.13	0.0
S05	2000	32.84 ± 0.33	32.68 ± 0.31	ND	787.82 ± 174.04	3642.68 ± 761.00	0.0
S06	2000	34.88 ± 0.86	39.23 ± 0.08	ND	142.61 ± 148.79	31.32 ± 27.18	0.0
S07	2000	34.81 ± 0.23	32.67 ± 0.31	ND	208.13 ± 33.67	3666.13 ± 762.15	0.0
S08	2000	33.12 ± 0.22	34.64 ± 0.54	ND	647.14 ± 92.86	1020.04 ± 370.59	0.0
S09	2000	32.50 ± 0.39	ND	ND	993.42 ± 263.45	0.0	0.0
S10	2000	ND	ND	ND	0.0	0.0	0.0
S11	2000	ND	ND	ND	0.0	0.0	0.0
S12	2000	ND	ND	ND	0.0	0.0	0.0
S13	2000	ND	ND	ND	0.0	0.0	0.0
S14	2000	ND	ND	ND	0.0	0.0	0.0
S15	2000	ND	37.04 ± 0.89	ND	0.0	222.96 ± 115.95	0.0
S16	2000	ND	36.94 ± 0.35	ND	0.0	216.87 ± 51.24	0.0
S17	2000	34.60 ± 0.61	34.13 ± 0.07	ND	180.49 ± 74.39	1303.99 ± 60.72	0.0
S18	2000	37.30 ± NA	38.15 ± 0.16	ND	8.82 ± 15.28	58.86 ± 51.42	0.0
S19	2000	32.32 ± 0.14	35.73 ± 0.38	ND	819.84 ± 83.38	454.29 ± 107.93	0.0
S20	2000	33.68 ± 0.27	36.13 ± 0.20	ND	324.65 ± 63.06	342.69 ± 45.91	0.0
S21	2000	33.90 ± 0.52	35.07 ± 0.22	ND	288.00 ± 100.37	696.64 ± 100.73	0.0
S22	2000	34.97 ± 0.75	31.02 ± 0.18	ND	142.51 ± 62.87	10,434.01 ± 1211.14	0.0
S23	2000	ND	39.18 ± 0.28	ND	0.0	29.82 ± 26.47	0.0
S24	2000	36.55 ± 1.91	36.75 ± 0.29	ND	66.53 ± 50.36	227.17 ± 44.06	0.0
S25	2000	ND	39.11 ± ND	ND	0.0	15.41 ± 26.69	0.0
S26	2000	31.41 ± 0.16	32.84 ± 0.21	ND	1538.87 ± 178.60	3099.79 ± 459.19	0.0
S27	2000	ND	ND	ND	0.0	0.0	0.0
S28	2000	31.02 ± 0.14	38.57 ± 1.38	ND	2015.58 ± 192.64	54.22 ± 66.16	0.0
S29	2000	28.85 ± 0.26	34.57 ± 0.48	ND	9086.65 ± 1727.22	1004.71 ± 334.88	0.0
S30	2000	ND	ND	ND	0.0	0.0	0.0
S31	2000	ND	ND	ND	0.0	0.0	0.0
S32	2000	ND	ND	ND	0.0	0.0	0.0

ND—not detected; NA—not available.

## Data Availability

The datasets generated and/or analyzed in the current study are available from the corresponding author upon reasonable request.

## Supplementary Materials

The following supporting information can be downloaded at https://www.mdpi.com/article/10.3390/biology15030219/s1: Figure S1: Multiple sequence alignment of the 28S rDNA region for *Alexandrium catenella*, *Alexandrium pacificum*, and *Gymnodinium catenatum* and their closely related species; Figure S2: Amplification curves from the specificity test of the multiplex qPCR assay for (A) *Alexandrium catenella* (blue, Cq = 18.340), (B) *Alexandrium pacificum* (red, Cq = 19.359), and (C) *Gymnodinium catenatum* (orange, Cq = 16.711). Non-target species (purple) and negative controls (gray) exhibited no amplification (Cq > 40). The threshold line indicates the fluorescence cutoff used for Cq determination; Table S1: Sampling information and locations of the 32 seawater stations; Table S2: List of non-target phytoplankton species used for specificity testing; Table S3: Detailed qPCR quantification results of environmental DNA samples.
