# Supplementary material for: Multiplex qPCR Assay for Simultaneous Detection of Three PST-Producing Dinoflagellates in the East China Sea off Southern Korea
Source: Biology (Basel). 2026 Jan 24;15(3):219. doi: 10.3390/biology15030219 (PMC12896616; doi:10.3390/biology15030219)
Supplement: Supplementary file 1 [file biology-15-00219-s001.zip › Supplementary_Figures.pdf]

Supplementary Materials

Multiplex qPCR Assay for Simultaneous Detection of Three PST-Producing Dinoflagellates in the East China Sea off Southern Korea

Jung Soo Heo [1,*], Biet Thanh Tran [1], Keun-Yong Kim [1], Sunju Kim [2], Seok Hyun Youn [3] and Tae Gyu Park [3]

[1] Bioinformatics Team, AquaGenTech Co., Ltd., Busan 48228, Republic of Korea; tranthanhbiet2502@gmail.com (B.T.T.); koby0323@hanmail.net (K.-Y.K.)
[2] Major of Oceanography, Division of Earth Environmental System Science, Pukyong National University, Busan 48513, Republic of Korea; sunkim@pknu.ac.kr
[3] National Institute of Fisheries Science, Busan 46083, Republic of Korea; younsh@korea.kr (S.H.Y.); taegyupark@korea.kr (T.G.P.)
* Correspondence: dgyjs2@daum.net, Tel.: +82-51-624-0307

Supplementary Figures

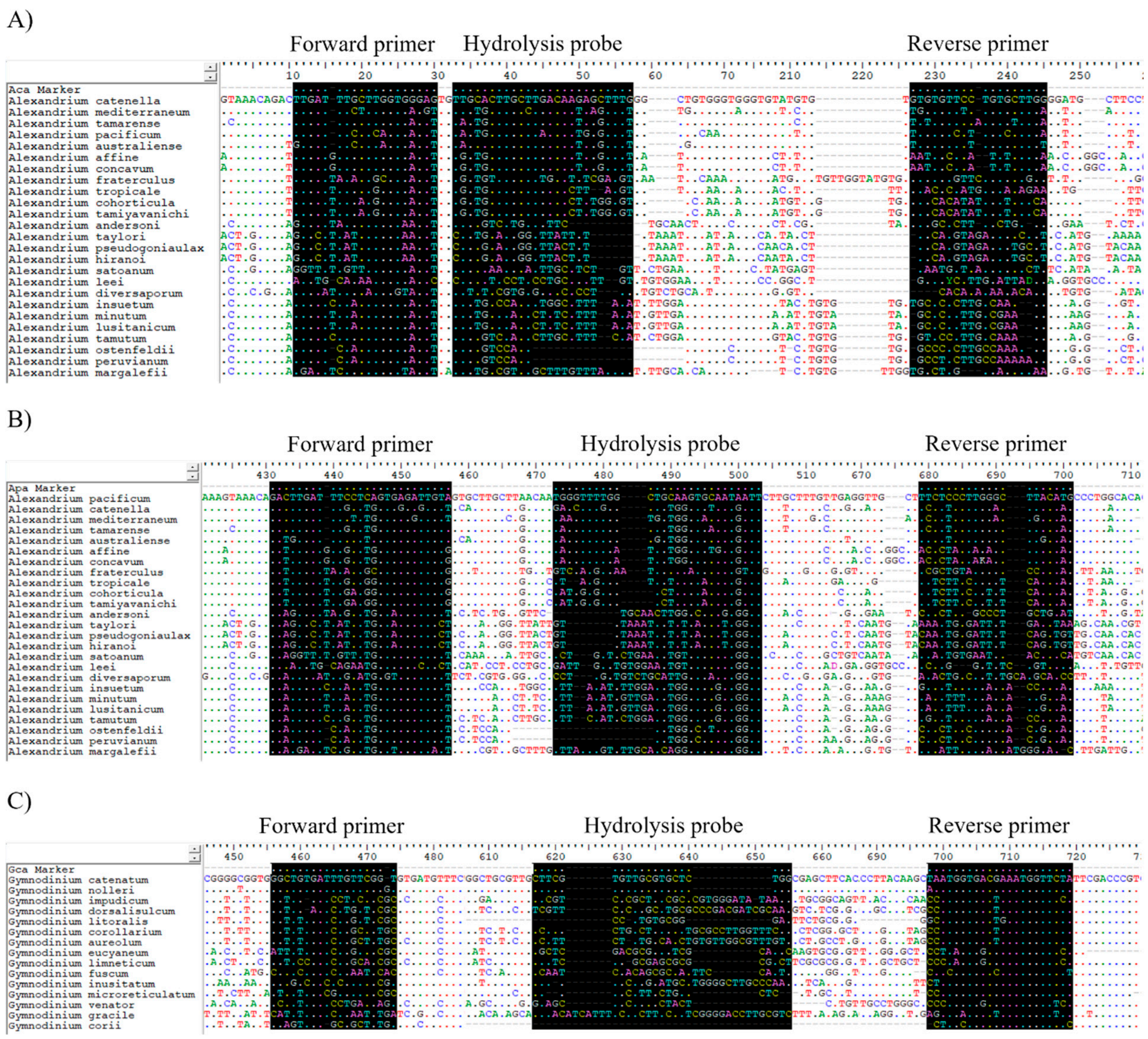

Figure S1. Multiple nucleotide sequence alignment of the 28S rDNA region for A) *Alexandrium catenella*, B) *Alexandrium pacificum*, and C) *Gymnodinium catenatum*, along with their congeneric species for their multiplex qPCR analysis. Conserved regions (black) were selected for designing species-specific primers and hydrolysis probe sets.

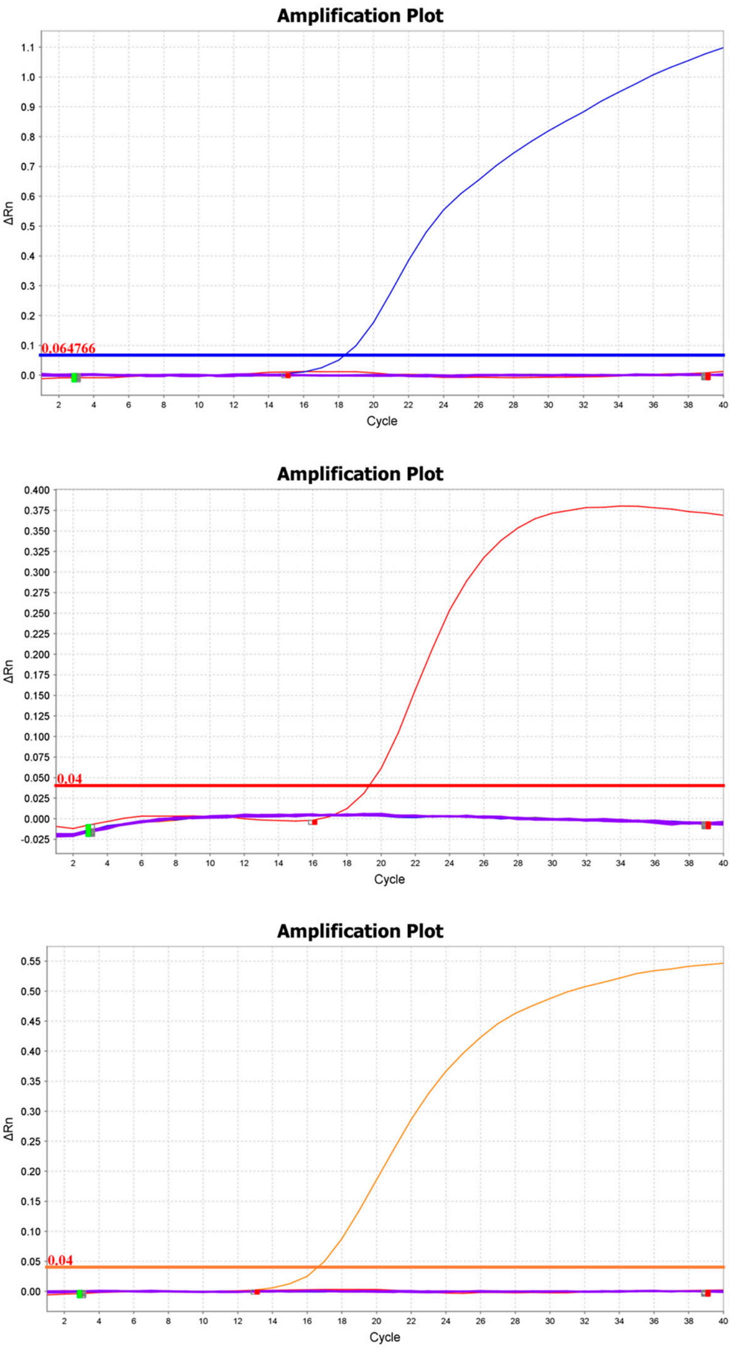

Figure S2. Amplification curves from the specificity test of the multiplex qPCR assay for (A) *Alexandrium catenella* (blue, $C_q = 18.340$), (B) *Alexandrium pacificum* (red, $C_q = 19.359$), and (C) *Gymnodinium catenatum* (orange, $C_q = 16.711$). Non-target species (purple) and negative controls (gray) exhibited no amplification ($C_q > 40$). The threshold line indicates the fluorescence cutoff used for C_q determination.
